# Dendritic cells a double-edge sword in autoimmune responses

**DOI:** 10.3389/fimmu.2012.00233

**Published:** 2012-08-02

**Authors:** Giada Amodio, Silvia Gregori

**Affiliations:** San Raffaele Telethon Institute for Gene Therapy (OSR-TIGET), Division of Regenerative Medicine, Stem Cells and Gene Therapy, San Raffaele Scientific Institute,Milan, Italy

**Keywords:** dendritic cells, autoimmune diseases, tolerance

## Abstract

Dendritic cells (DC) are antigen-presenting cells that play a pivotal role in regulating innate and adaptive immune responses. In autoimmunity, DC act as a double-edged sword since on one hand they initiate adaptive self-reactive responses and on the other they play a pivotal role in promoting and maintaining tolerance. Thus, DC are the most important cells in either triggering self-specific responses or in negatively regulating auto-reactive responses. The latter function is mediated by DC in the steady-state or specialized subsets of DC, named tolerogenic DC. Clinical and experimental evidence indicate that prolonged presentation of self-antigens by DC is crucial for the development of destructive autoimmune diseases, and defects in tolerogenic DC functions contribute to eradication of self-tolerance. In recent years, DC have emerged as therapeutic targets for limiting their immunogenicity against self-antigens, while tolerogenic DC have been conceived as therapeutic tools to restore tolerance. The purpose of this review is to give a general overview of the current knowledge on the pathogenic role of DC in patients affected by autoimmune diseases. In addition, the protective role of tolerogenic DC will be addressed. The currently applied strategies to block immune activation or to exploit the tolerogenic potential of DC will be discussed.

## INTRODUCTION

Dendritic cells (DC) are professional antigen-presenting cells (APC) specialized in capturing and processing antigens (Ags) to present to T cells. DC constitute a front-line defense against pathogens, are located throughout the body, and form complex networks that allow them to communicate with different cells. Therefore, DC are critically involved in the initiation of adaptive immune responses and, as such, are defined immunogenic DC. These DC might be implicated in the induction of autoimmune responses *via* the activation of auto-reactive T cells and the consequent eradication of self-tolerance. Conversely, DC in the steady-state, or specialized subsets of DC, termed tolerogenic DC, promote and maintain tolerance through several non-overlapping mechanisms. Tolerogenic DC can induce apoptosis of effector T cells, skew T cell phenotype, and promote anergy and/or regulatory T cells (Tregs; Morelli and Thomson, 2007; Gregori, 2011). Thus, defects in the activities of tolerogenic DC may also contribute to break self-tolerance and to induce autoimmune responses.

An optimal balance between immunogenic and tolerogenic DC is therefore fundamental to prevent self-reactive immune responses and to maintain immune self-specific homeostasis. In this review, we will give an overview of the different role of both immunogenic and tolerogenic DC in promoting autoimmune disease onset and/or progression, focusing primarily on human pathological conditions.

## HUMAN DENDRITIC CELL SUBSETS

Dendritic cells are present in all tissues and they function as an important bridge between innate and adaptive immunity, by cellular interactions or through secretion of pro-inflammatory and immuno-regulatory cytokines (Banchereau and Steinman, 1998; Larregina and Falo, 2005; Merad et al., 2008; Rescigno and Di Sabatino, 2009; Lambrecht and Hammad, 2010; Thomson, 2010).

In the bloodstream, DC circulate as immature cells characterized by a low expression of human leukocyte antigen (HLA) class II and co-stimulatory molecules, high endocytic activity, and low T cell activation potential. Circulating DC constantly patrol the surrounding environment for pathogens, such as viruses and bacteria. Upon Ag encounter, DC undergo a complex process of maturation meanwhile they travel to the lymph nodes, where they activate helper and cytotoxic T cells as well as B cells. Immature DC in the steady-state migrate at low ratio to the lymph nodes without undergoing activation, can present Ags to T cells in the absence of co-stimulation and induce clonal T cell anergy (Schwartz et al., 1989), deletion of auto-reactive T cells (Hawiger et al., 2001; Steinman and Nussenzweig, 2002), and promote Tregs (Dhodapkar et al., 2001). Tolerogenic DC, both circulating and tissue resident, contribute to the induction and maintenance of self-specific tolerance.

In humans, two major and intrinsically different subpopulations of DC have been described: myeloid DC (myDC), called also conventional DC, and plasmacytoid DC (pDC), which differ in their transcriptional program, development, phenotypic markers, and immunological functions (Belz and Nutt, 2012). myDC pick up Ags in the periphery and move to T cell areas of peripheral lymphoid organs to initiate immunity through a number of different events including maturation and cytokine secretion, all of which are regulated by recognition of pathogens *via* Toll-like receptors (TLR; Watts et al., 2010).

Myeloid DC are present in the peripheral blood and in several tissues where they acquire specialized functions. In the bloodstream, several subpopulations of immunogenic myDC, all of them expressing CD11c, and the myeloid markers CD13 and CD33, are present (**Table 1**). These cells include CD16^+^ (they are also characterized by the expression of M-DC8; Schakel et al., 1999), BDCA-1^+^, and BDCA-3^+^ (Dzionek et al., 2001) that have different ability to stimulate allogeneic T cells (MacDonald et al., 2002). Distinct phenotypical and functional characteristics are displayed by myDC resident in peripheral tissues. These myDC can be distinguished according to the expression of specific markers: langerin (CD207) expressing cells (Geissmann et al., 2002; Larregina and Falo, 2005) are Langerhans cells (LC) and interstitial dermal DC localized in the skin; CD103^+^ DC reside in the lamina propria (LP) of the small intestine (Jaensson et al., 2008; Rescigno and Di Sabatino, 2009); C-type lectin^+^ (DC-SIGN) DC are present in the decidua (Laskarin et al., 2007); BDCA-1^+^ and BDCA-3^+^ DC have been described in the lung (Demedts et al., 2005; **Table 1**).

**Table 1:** Different subsets of human dendritic cells.

Tissue distribution Markers			Reference
**Myeloid DC Immunogenic**			
BDCA-1	Blood/tissues	CD11c, BDCA-1	MacDonald etal. (2002), Dzionek etal. (2000), 2001
BDCA-3	Blood/tissues	CD11c, BDCA-3	MacDonald etal. (2002), Dzionek etal. (2000), 2001
M-DC8	Blood/tissues	M-DC8, CD16	Schakel etal. (1999)
DC-SIGN	Blood/decidua	CD11c, DC-SIGN	Laskarin etal. (2007)
Langerhans cells	Skin	CD207, CD1a	Larregina and Falo (2005), Geissmann etal. (2002)
**Myeloid DC tolerogenic**			
CD163	Blood	CD11c, ILT3	Maniecki etal. (2006)
DC-10	Blood/tissues	CD11c, CD14, CD16, CD83, HLA-G, ILT4	Gregori etal. (2010)
CD103	Lamina propria	CD11c, CD103	Jaensson etal. (2008), Rescigno and Di Sabatino (2009)
**Plasmacytoid DC**			
pDC	Blood/tissues	BDCA-2, BDCA-4, CD123, ILT7	Dzionek etal. (2000), 2001, Jahnsen etal. (2000), Cao and Bover (2010)

In addition to immunogenic myDC, other subsets of myDC with tolerogenic properties have been described such as DC expressing the scavenger receptor CD163 and immunoglobulin-like transcript 3 (ILT3; Maniecki et al., 2006). We recently identified DC-10, which are tolerogenic DC characterized by the expression of CD11c^+^, CD14^+^, CD16^+^, CD83^+^, and the tolerogenic molecules HLA-G and ILT4 (Gregori et al., 2010). DC-10 display a mature phenotype since they express both HLA class II and co-stimulatory molecules. They have a unique cytokine secretion profile consisting of high levels of IL-10 in the absence of IL-12 (Gregori et al., 2010). Specialized subsets of tolerogenic DC have been described in each tissue where they maintain tissue homeostasis and tolerance (reviewed in Gregori, 2011).

Plasmacytoid DC are component of the innate immune system and are specialized in producing interferon-α (IFN-α) upon activation *via* TLR7- and TLR9-mediated recognition of nucleic acids, and participate in T cell immunity (reviewed by Colonna et al., 2004). Similar to myDC, immature pDC as well as alternatively activated pDC are involved in promoting tolerance (Hanabuchi et al., 2010; Martin-Gayo et al., 2010). pDC are characterized by the expression of BDCA-2, BDCA-4 (Dzionek et al., 2001), IL-3R (CD123; Jahnsen et al., 2000), and ILT7 (Cao and Bover, 2010). pDC are found in the peripheral blood, lymph nodes, and the thymus, and they are recruited to sites of inflammation under pathological conditions (Swiecki and Colonna, 2010).

## DENDRITIC CELLS IN CENTRAL AND PERIPHERAL TOLERANCE

To avoid autoimmune reactions, self-reactive lymphocytes have to be deleted or rendered tolerant. Several mechanisms are operating in the central and peripheral compartments to induce and maintain tolerance. Defects in these mechanisms are associated with the activation of immune responses against self-Ags (Goodnow et al., 2005). Central tolerance occurs in the thymus and leads to the deletion of self-reactive T cells through the positive and negative selection (Hogquist et al., 2005). The role of DC in central tolerance has become evident in the last decades. Thymic myDC are very efficient in mediating negative selection of developing thymocytes (Brocker et al., 1997; Ohnmacht et al., 2009). In addition, peripheral myDC can migrate to the thymus and contribute to negative selection (Bonasio et al., 2006; Proietto et al., 2008). Both thymic myDC and pDC play an important role in promoting positive selection of Tregs (Proietto et al., 2008; Hanabuchi et al., 2010; Martin-Gayo et al., 2010). Thus, myDC and pDC cooperate in the thymus to promote on one hand negative selection of self-reactive T cells, and on the other positive selection of Tregs.

To control immune responses to self-Ags that are not expressed in the thymus or may escape negative selection, different mechanisms of tolerance are operational in the periphery during the entire lifespan. Mechanisms of peripheral tolerance include cell death with consequent clonal deletion, development of a state of T cell unresponsiveness, and active suppression mediated by Tregs. DC, *via* the production of the immuno-modulatory cytokines IL-10 and TGF-β or the expression of the tolerogenic molecules indoleamine 2,3-dioxygenase (IDO) or ILTs (Morelli and Thomson, 2007; Gregori, 2011), can regulate several of these processes.

## ROLE OF DENDRITIC CELLS IN PRIMING AND SUSTAINING SELF-REACTIVE IMMUNE RESPONSES

In genetically susceptible individuals, autoimmune diseases may develop as a result of alterations in the expression of self-Ags by DC, or access to immune privileged sites, or modification of the activation state of DC that became potent activators/inducers of self-reactive effector T cells. Multiple evidences from pre-clinical models of autoimmune diseases indicate that DC loaded with self-Ags acquired an activated phenotype and are able to trigger autoimmune responses *via* the induction of T helper 1 (Th1) and Th17 responses (Torres-Aguilar et al., 2010). Priming of self-reactive T cells by activated DC that have taken up apoptotic cell debris may also lead to break-down self-tolerance and can result in autoimmunity (Lleo et al., 2008). The pro-inflammatory environment generally observed in organs target of autoimmunity can modify several tolerogenic DC functions, shifts the balance between tolerogenic and immunogenic DC toward the latter, and contributes to the development of autoimmune diseases.

Several factors in autoimmune patients indicate that the dysregulation in the immunogenic and tolerogenic DC is associated with excessive self-reactive responses and inflammation.

### ABERRANT ACTIVATION OF IMMUNOGENIC DENDRITIC CELLS IN HUMAN AUTOIMMUNE DISEASES

In the last decades, studies of DC in patients indicate that aberrant DC activation or functions are associated with different autoimmune diseases as including Rheumatoid Arthritis (RA), Multiple Sclerosis (MS), Systemic Lupus Erythematosus (SLE), Psoriasis, and Inflammatory Bowel Disease (IBD; **Table 2**).

**Table 2:** Role of dendritic cell subsets in human autoimmune diseases.

DC subsets	Localization	Function	Reference
Rheumatoid Arthritis	myDC	Blood/synovial tissues	↑ Effector T cell priming	Santiago-Schwarz (2004),
			↑ Pro-inflammatory cytokines	Takakubo etal. (2008)
			↑ DC activation	
	pDC	Synovial tissues	↑ IFN-α production	Takakubo etal. (2008),
			↑ B cell activation	Lebre etal. (2008)
Multiple Sclerosis	myDC	Cerebrospinal fluid	↑ T cell activation	Pashenkov etal. (2002),
			↑ Pro-inflammatory cytokines	Wu and Laufer (2007),
				Karni etal. (2006)
	pDC	Blood	Impairment in maturation	Stasiolek etal. (2006)
			↓ Ability to prime FOXP3^+^ Tregs	
Systemic Lupus Erythematosus	myDC	Blood	↑ DC activation	Seitz and Matsushima (2010),
			↑ Effector Th1/Th17 cell priming	Fransen etal. (2009)
	pDC	Inflamed lesions/LN	↑ Effector T cell priming	Robak etal. (2004),
				Migita etal. (2005),
				Gerl etal. (2010)
Psoriasis	myDC	Psoriatic lesions	↑ Pro-inflammatory cytokine	Lowes etal. (2005),
			↑ Effector T cell priming	Zheng etal. (2007)
	pDC	Psoriatic lesions	↑ IFN-α production	Nestle etal. (2005)
Inflammatory Bowel Disease****	myDC	Inflamed lesions	↑ DC activation	te Velde etal. (2003),
			↑ Pro-inflammatory cytokines	Hart etal. (2005)
			↑ T cell activation	
	pDC	Lamina propria	↑ DC activation	Baumgart etal. (2011)
			↑ IFN-α production	
			↓ TNF-α production	

#### Rheumatoid Arthritis

In the peripheral blood of RA patients, but also in synovial fluids and tissues, increased numbers of myDC and pDC are present (Lebre and Tak, 2009). Studies on myDC from synovial fluid of RA patients show that these cells display an activated phenotype as they express high levels of HLA-DR and co-stimulatory molecules. Interestingly, myDC in inflamed tissues are associated with T cells in structures similar to germinal centers where they stimulate self-reactive T cells (Santiago-Schwarz, 2004). These myDC are also involved in promoting synovial inflammation due to their ability to secrete pro-inflammatory cytokines (Jongbloed et al., 2006; Lebre et al., 2008).

The role of pDC in the RA pathogenesis is dual: on one hand in synovial tissues pDC *via* the secretion of type I IFNs contribute to local inflammation, although at lower extend as compared to myDC (Pettit et al., 2000; Takakubo et al., 2008); on the other hand, pDC could play a role in activating B cells *via* the expression of B cell-activating factor (Lebre et al., 2008), leading to antibody production, which sustain tissue damage.

#### Multiple Sclerosis

The active participation of DC in the MS pathology is supported by their presence and activation in the central nervous system (CNS) of MS patients (Pashenkov et al., 2002). Increased frequency of myDC in the CNS at early stages of the disease and their presence within the demyelinating lesions indicate that myDC play a role in re-activating T cell responses to myelin upon entry into the CNS (Wu and Laufer, 2007). In addition to their identification in the CNS during the disease, analyses of myDC in the peripheral blood of MS patients revealed their ability to secrete pro-inflammatory cytokines at higher levels than DC from normal donors (Karni et al., 2006). These activated myDC, polarize CD4^+^ T cells toward IFN-γ-producing effector cells (Karni et al., 2006; Vaknin-Dembinsky et al., 2006). Thus, myDC in MS patients are highly immunogenic and contribute to disease induction and progression.

The role of pDC in the MS pathogenesis is less clear. No differences in the absolute number of pDC have been found in the peripheral blood of MS patients. However, a reduced stimulatory activity of pDC and a limited expression of co-stimulatory molecules upon *in vitro* activation were described, suggesting an impairment in the maturation and an altered regulatory functions of pDC in MS patients (Stasiolek et al., 2006).

#### Systemic Lupus Erythematosus

The induction of SLE and disease severity is associated with a defect in clearance of apoptotic cells by macrophages (Herrmann et al., 1998). This results in hyper-activation of DC and leads to the chronic inflammation observed in SLE (Seitz and Matsushima, 2010). When apoptotic cells are not rapidly removed, they release blebs, in which SLE auto-Ags are clustered, and induce maturation of DC. These DC can stimulate the production of IL-2, IFN-γ, and, in particular, IL-17 by T cells that sustain autoimmune responses (Fransen et al., 2009). Although myDC are reduced in the peripheral blood of SLE patients (Robak et al., 2004; Migita et al., 2005), they contribute to effector T cell activation because of their activated phenotype (Ding et al., 2006; Gerl et al., 2010). In line with this notion, monocytes from SLE patients undergo an accelerated differentiation *in vitro* and express high levels of co-stimulatory molecules (Ding et al., 2006; Gerl et al., 2010).

Plasmacytoid DC are also reduced in the peripheral blood of SLE patients (Robak et al., 2004; Migita et al., 2005), but they accumulate in inflamed skin lesions (Mori et al., 1994) where they are selectively attracted by ChemR23, the chemokine receptor for chemerin (Vermi et al., 2005). Moreover, circulating pDC from SLE patients migrate in response to CCL19 (Gerl et al., 2010). It has been proposed that the increased responsiveness to CCL19 might lead to pDC accumulation in T cell area of lymph nodes where they increase the priming of self-reactive T cells and contribute to SLE pathogenesis (Gerl et al., 2010).

#### Psoriasis

In psoriatic lesions, the frequency of myDC is 30-fold increased with respect to normal skin (Zaba et al., 2007). The large proportion of these cells secretes TNF-α, IL-12, IL-23, and the inducible nitric oxide synthase (iNOS; Lowes et al., 2005). These cytokines activate keratinocytes and fibroblasts to secrete pro-inflammatory cytokines (IL-6 and IL-1) that induce effector Th1 and Th17 cells, contributing to dermal inflammation and epidermal hyperplasia characteristic of psoriasis (Zheng et al., 2007; Pene et al., 2008).

The frequency of IFN-α-secreting pDC is also increased in psoriatic lesions and participate to local inflammation (Nestle et al., 2005).

#### Inflammatory Bowel Disease

Several studies in Crohn’s disease (CD) and ulcerative colitis (UC) patients have demonstrated an abnormal intestinal accumulation of DC expressing BDCA-1, which contribute to excessive T cell activation (de Baey et al., 2003; te Velde et al., 2003; Silva et al., 2004). DC from CD patients have an altered cytokine production profile since they produce higher levels of IL-12 and IL-6 than DC from healthy donors (Hart et al., 2005). Thus, myDC accumulate in the intestine of IBD patients where they activate pathogenic T cells.

It has been recently reported that pDC might participate to inflammation in the mucosa of CD and UC patients. Indeed high frequency of pDC was found in inflamed mucosa of CD and UC patients. Studies on pDC from the peripheral blood of flaring CD and UC patients demonstrated that they express higher levels of CD40 and CD86, and they secrete higher amounts of TNF-α than pDC from healthy subjects. However, these pDC were impaired in their ability to secrete IFN-α (Baumgart et al., 2011). Thus, these results suggest that aberrant activation of pDC or alteration in their regulatory functions could play a role in the pathogenesis of IBD.

These examples clearly indicate that hyper-activation of myDC is one of the key factors in promoting self-reactive T cell immunity. Moreover, an aberrant pDC distribution and function contribute to the local inflammation in target organs of autoimmunity. In this scenario, activated DC are recruited to the inflamed tissues where they secrete pro-inflammatory cytokines (i.e., IL-1, TNF-α, IFN-α, and IL-6) or express high levels of co-stimulatory molecules that induce an immune-stimulatory loop causing re-activation of self-reactive T cells and recruitment and/or the activation of other immune cells, including additional DC.

### ALTERATION OF TOLEROGENIC DENDRITIC CELL FUNCTIONS AND AUTOIMMUNITY

In homeostatic and resting conditions (in the absence of inflammation) DC preserve an immature or semi-mature phenotype, and actively participate in the maintenance of tolerance toward self-Ags. In these conditions, tissue resident tolerogenic DC control self-reactive T cell responses by preventing excessive local inflammation and autoimmune-mediated tissue damages. The presence of high levels of pro-inflammatory mediators observed in chronic inflamed tissues decreases the regulatory activity of tolerogenic DC.

One of the most important features of tolerogenic DC is their ability to secrete immuno-regulatory cytokines, such as IL-10 and TGF-β. IL-10 directly suppresses T cell responses by inhibiting the secretion of IL-2 and IFN-γ (Vieira et al., 1991) and by preventing T cell proliferation (Taga and Tosato, 1992). Similarly, TGF-β potently inhibits T cell responses (Gorelik and Flavell, 2002). IL-10 controls a number of different cells implicated in inflammatory responses, including APC (Mosser and Zhang, 2008). The expression of HLA class II, co-stimulatory molecules (de Waal Malefyt et al., 1991) and pro-inflammatory cytokines (Fiorentino et al., 1991a, b) is down-regulated by IL-10. On the other hand, IL-10 up-regulates the expression of tolerogenic molecules such as ILT3 and ILT4 (as reviewed in Suciu-Foca et al., 2005), and HLA-G (Moreau et al., 1999) on APC, rendering them capable of dampening immune responses and inducing Tregs (Carosella et al., 2011). In the steady-state, DC secrete high levels of IL-10, can modulate the activation of neighboring myDC, and promote the *de novo* induction of tolerogenic DC. *In vitro* studies demonstrated that maturation of monocytes derived DC in the presence of exogenous IL-10 is inhibited, and resulting DC become able to induce anergic/suppressive T cells (Steinbrink et al., 1997, 2002). Moreover, differentiation of monocytes derived DC in the presence of IL-10 results in a population of mature myDC, called DC-10, which secrete high levels of IL-10 and are potent inducers of Ag-specific IL-10-producing type 1 regulatory (Tr1) cells *in vitro* (Gregori et al., 2010; Pacciani et al., 2010). In addition to their ability to secrete high levels of IL-10, DC-10 strongly express ILT4 and HLA-G, which are necessary for efficient Tr1 cell induction. In inflamed tissues, high amounts of pro-inflammatory cytokines lead to the down-regulation of IL-10 production that could impair the modulation of already differentiated DC, and the *de novo* induction of tolerogenic DC, including DC-10.

It has been reported that mutations in IL-10 or in its receptor lead to the loss of IL-10 function and cause severe intractable infant and adult enterocolitis (Glocker et al., 2009, 2010), demonstrating the critical role of IL-10 in maintaining intestinal tolerance. More recently, it has been shown that DC generated from peripheral monocytes of IBD children carrying a mutation in IL-10R secrete significantly higher amounts of TNF-α, IL-12, and IL-23 than DC from healthy controls (Begue et al., 2011). These data indicate that impairment in the ability of DC to produce IL-10 and to respond to it is critically involved in the pathogenesis of IBD.

In addition to soluble factors, tolerogenic DC can express immuno-regulatory enzymes such as IDO and heme oxygenase-1 (HO-1), which suppress T cell responses and promote immune tolerance. IDO inhibits effector T cell proliferation by reducing tryptophan that is necessary for cell division (Mellor and Munn, 2004). HO-1 is the rate-limiting enzyme in heme catabolism and it acts as an anti-inflammatory molecules, controlling apoptosis, T cell proliferation and activation (Otterbein et al., 2000; Pae et al., 2004). In non-pathological conditions, Foxp3^+^ Tregs promote IDO expression in myDC through the interaction of cytotoxic T-lymphocyte antigen 4 (CTLA-4) with CD80 and CD86 (Fallarino et al., 2002, 2003; Grohmann et al., 2002). Resulting myDC acquire the ability to generate Foxp3^+^ Tregs (Mellor and Munn, 2004). During inflammation, chronically activated myDC, although expressing high levels of CD80 and CD86, become refractory to the inhibitory signal induced by Foxp3^+^ Tregs and unable to express IDO.

Indoleamine 2,3-dioxygenase can also be expressed by pDC alternatively activated with anti-CD40L and IL-3 (Martin-Gayo et al., 2010) or with thymic stromal lymphopoietin (TSLP; Hanabuchi et al., 2010). These IDO expressing pDC have been shown to promote the induction of Foxp3^+^ Tregs. In the synovial fluid of RA patients, IDO expressing pDC have been identified (Takakubo et al., 2008), but their limited number and the presence of an increased frequency of activated myDC impair their ability to counteract self-reactive effector T cell responses by the induction of Tregs.

Immune cells and non-immune cells can play an important role in driving the development of tolerogenic DC. It has been shown that human intestinal epithelial cells (IECs) through the secretion of TSLP, TGF-β, and retinoic acid drive the development of CD103^+^ tolerogenic DC (Iliev et al., 2009). CD103^+^ DC promote the *de novo* induction of Foxp3^+^ Tregs and inhibit Th1 and Th17 responses (Iliev et al., 2009). In CD patients, IECs do not express TSLP and fail to control DC-mediated pro-inflammatory responses, resulting in abnormal release of IL-12 (Rimoldi et al., 2005) and reduced ability to induce CD103^+^ DC (Rescigno and Di Sabatino, 2009). This perturbation in the cross-talk between IECs and DC disrupts the intestinal immune-homeostasis and promotes gut inflammation.

In conclusion, chronic inflammation and the presence of high levels of pro-inflammatory cytokines in target organs of autoimmunity and in the periphery alters the regulatory activity of tolerogenic DC and generate an imbalance between tolerogenic and immunogenic DC, which sustains constant activation of self-reactive T cells leading to tissue damage.

## STRATEGIES TO PROMOTE TOLERANCE BY TARGETING DENDRITIC CELLS

Autoimmune diseases are the result of a potent and de-regulated immuno-responses toward self-Ags mediated by a variety of immune cells, including B and T lymphocytes, and APC. The critical role of DC in the initiation and in the progression of autoimmune diseases indicates that DC targeting therapies could represent a good alternative to current immuno-modulatory therapies already approved for the treatment of autoimmune diseases. Two alternatives approaches can be foreseen to modulate DC: (i) therapies targeting immunogenic DC to lower their activation, (ii) therapies targeting tolerogenic DC to improve their function and induction.

Treatment with monoclonal antibodies (mAb) against pro-inflammatory cytokines or their receptors aiming to reduce the DC immunogenicity are currently under clinical investigation for the treatment of autoimmune diseases. Administration of Anakinra, a recombinant version of IL-1Rα, in combination with methotrexate (MTX), or of Tocilizumab, a humanized mAb that competes with IL-6 for receptor binding, provided good clinical benefit in RA patients (Smolen et al., 2008; Niu et al., 2011). Positive results were obtained also in patients with RA, CD, and psoriasis treated with anti-TNF-α mAb (Infliximab; Present et al., 1999; Cohen et al., 2000; Ricart et al., 2001). Two recent phase II clinical trials, proved the efficacy and safety of a two different mAbs against IL-17 (Ixekizumab; Leonardi et al., 2012) or its receptor (Brodalumab; Papp et al., 2012) for the treatment of Psoriasis. Despite these encouraging results, additional studies are needed to evaluate the safety of long-term treatment with these mAbs and to define the optimal schedule for their efficacy. Notably, to obtain stable clinical benefit, chronic administration of these mAbs is required since clinical symptoms return after treatment withdrawal.

An alternative approach to block DC immuno-stimulatory activity is the inhibition of co-stimulatory molecules (CD80 and CD86). In pre-clinical models of autoimmune diseases the efficacy of CD28/B7 blockade by CTLA-4Ig has been shown (Salomon and Bluestone, 2001). Interestingly, while the efficacy and tolerability of CTLA-4Ig (Abatacept) have been reported across multiple international, randomized, double blind, placebo control trials in patients with active RA (Massarotti, 2008), its effect in other autoimmune diseases, such as Psoriasis and MS, is still not clear (Sakthivel, 2009) and additional investigations are required.

Results from these clinical trials indicate that therapies with mAb aim at inhibiting pro-inflammatory cytokines or co-stimulatory signaling pathways are efficacious; however, they required long-term administration with consequent long-term detrimental effects for patients.

Another alternative strategy to restore tolerance in autoimmunity is to improve the induction and function of tolerogenic DC. The majority of the efforts have been focused on generating tolerogenic DC *in vitro* to be subsequently administered *in vivo* as cell therapy, rather than in promoting *in vivo* the expansion of tolerogenic DC. Different immune-modulatory agents have been used in order to modify the phenotype, cytokine profiles and activity of DC. Encouraging results have been obtained by treating DC with biological agents such as dexamethasone (Piemonti et al., 1999) or vitamin D3 (Penna and Adorini, 2000) or cytokines such as TNF-α (van Duivenvoorde et al., 2004, 2007) or IL-10 (Steinbrink et al., 1997, 2002; Sato et al., 2003; Gregori et al., 2010). In pre-clinical models of arthritis (van Duivenvoorde et al., 2004, 2007), EAE (Menges et al., 2002), and type 1 diabetes (T1D; Feili-Hariri et al., 2002) the efficacy of *in vitro* induced tolerogenic DC-based cell therapy has been demonstrated. In addition, repetitive injection of immature DC has been shown to protect mice from collagen-induced arthritis (Charbonnier et al., 2006). To date, in the field of autoimmune diseases, no data have been published using immuno-modulatory pDC as therapeutic tools.

Despite the fact that *in vitro* generated human tolerogenic DC have been studied in research settings, the described methods have not been translated into clinical grade protocols. Recently a comparative analysis of good manufacturing practice protocols to generate human tolerogenic DC using IL-10, TGF-β, vitamin D3, dexamethasone or rapamycin has been performed (Boks et al., 2012). Results from this study demonstrated that DC activated in the presence of IL-10 (IL-10 DC) showed the most powerful tolerogenic characteristics with high IL-10 production and low T cell activation. Based on these results the authors suggested that IL-10 DC are the best suitable subset of tolerogenic DC for tolerance inducing therapies. We developed a protocol to generate human tolerogenic DC by differentiating monocyte derived DC in the presence of exogenous IL-10. Resulting cells, called DC-10, represent a powerful subset of tolerogenic DC. DC-10 are phenotypically and functionally stable and upon activation they maintain their cytokine production profile (high IL-10/IL-12 ratio) and their ability to differentiate adaptive Ag-specific Tr1 cells (S. Gregori, personal communication). In alternative to IL-10, a method to generate clinical grade tolerogenic DC from patients with RA using vitamin D3 and dexamethasone has been also developed (Harry et al., 2010) and a clinical trial for treating RA patients will be initiated soon (Moreau et al., 2009). Results from this first proof of principle clinical trial will provide informations on the safety and efficacy of tolerogenic DC-based cell therapy to restore tolerance in autoimmune settings.

## CONCLUSIONS AND PERSPECTIVES

Over the past years significant progresses have been achieved in understanding the pathological role of DC in autoimmune diseases and how tolerogenic DC regulate and maintain tolerance toward self-Ags. Although a number of questions still remain to be addressed, inhibition of the immunogenic branch of DC function or induction of the tolerogenic one has become a feasible approach to restore tolerance in autoimmune diseases. Current approaches based on the administration of mAb against immunogenic proteins have been successful, however the lack of information regarding long-term safety and the chronic infusion limited their broaden application. Alternatively, *in vitro* differentiated tolerogenic DC are of great potential interest as cell therapy for there-establishment of immunological tolerance in autoimmune diseases. Nevertheless, the optimal type of tolerogenic DC still remains to be defined. It has to be taken into account that tolerogenic DC should be resistant to maturation either induced by *in vivo* transfer or by inflammatory mediators. Moreover, the route and dose of administration as well as the need of *in vivo* pharmacological treatments for maintaining their tolerogenic functions have to be still determined. Further studies in humanized mouse model as well as in large animals will elucidate these aspects and will allow the establishment of protocols with tolerogenic DC-based cell therapy for clinical application in autoimmune diseases.

## Conflict of Interest Statement

The authors declare that the research was conducted in the absence of any commercial or financial relationships that could be construed as a potential conflict of interest.
